# Recurrent bacteremia after injection of *N*-butyl-2-cyanoacrylate for treatment of bleeding gastric varices: a case report and review of the literature

**DOI:** 10.1186/s13104-015-1679-6

**Published:** 2015-11-19

**Authors:** Bruno A. Randi, Daniel A. Ninomiya, Elizabeth L. Nicodemo, Beatriz C. Lopes, Eduardo R. Cançado, Anna S. Levin

**Affiliations:** Division of Infectious and Parasitic Diseases, Clinical Hospital, University of São Paulo Medical School, Dr. Ovídio Pires de Campos Street, 225—Cerqueira César, São Paulo, SP 05403-010 Brazil; Department of Gastroenterology, University of São Paulo School of Medicine, Dr. Ovídio Pires de Campos Street, 225—Cerqueira César, São Paulo, SP 05403-010 Brazil

**Keywords:** Cyanoacrylates, Esophageal and gastric varices, Bacteremia, Liver cirrhosis

## Abstract

**Background:**

Bleeding from gastric varices has high mortality rate, and obliteration using *N*-butyl-2-cyanoacrylate is the treatment of choice. Recurrent bacteremia is rarely reported following the procedure. We aimed to report a case of recurrent bacteremia after *N*-butyl-2-cyanoacrylate treatment and to review published cases.

**Case presentation and review:**

In May 2014, a 43-year-old Brazilian male presented with lower gastrointestinal bleeding. Endoscopy showed active bleeding from gastric varix. Injection of *N*-butyl-2-cyanoacrylate was performed and the patient was discharged. Over the next 4 months he presented with three episodes of bacteremia with severe sepsis and no identifiable focus of infection. Oral prophylaxis was initiated in September 2014 and he has remained free of bacteremia. Six other cases of recurrent bacteremia following sclerosis with *N*-butyl-2-cyanoacrylate were reported in the literature. All patients had portal hypertension and bleeding from gastric varices. Average age of patients was 55.7 years and the median time from endoscopic procedure to the first episode of bacteremia was 105 days (range 14–365). The mean number of episodes of bacteremia per patient was 2.5.

**Conclusion:**

Recurrent bacteremia associated with endoscopic treatment with *N*-2-butyl-cyanoacrylate is rare, but should be suspected in patients in which investigation shows no other focus of infection. Secondary prophylaxis should be considered after the first episode.

## Background

Prevalence of gastric varices in patients with portal hypertension varies from 18 to 70 % [1]. Bleeding from gastric varices has a mortality rate of 45 % [1]. There are many treatment options for bleeding gastric varices and, although limited data exist on optimal management, the first-line treatment advocated is endoscopic obliteration using *N*-butyl-2-cyanoacrylate. This liquid glue polymerizes immediately into a plastic cast after contact with blood [2, 3].

Bacteremia after endoscopic procedures may occur in a frequency of 4.2 %, and can be higher in patients with chronic liver disease and in therapeutic procedures [3]. Transient bacteremia following injection of cyanoacrylate is not uncommon [2], but recurrent bacteremia is rarely reported. We aimed to report a case of recurrent bacteremia after *N*-butyl-2-cyanoacrylate treatment and to review the literature for published cases.

## Case report

A 43-year-old Brazilian male was under follow-up at the gastroenterology outpatient clinic due to autoimmune hepatitis-associated liver cirrhosis (Child-Pugh class A/MELD 13). He had portal hypertension and diabetes mellitus and used azathioprine (100 mg/day) and prednisone (10 mg/day). He presented at our emergency department with lower gastrointestinal bleeding in May 2014. Upper endoscopy showed active bleeding from a gastric varix. Hemostasis was achieved by injection of *N*-butyl-2-cyanoacrylate and the patient was discharged 3 days after admission, using propranolol. In July 2014, the patient returned to our hospital with a history of 7 days of fever (up to 40 °C) associated with tachycardia (cardiac rate: 110 beats/min) and hypotension (blood pressure: 80/50 mmHg). There was no headache, nausea, vomiting, diarrhea, abdominal pain, cough, urinary symptoms or bleeding. The patient was admitted to the intensive care unit and ceftriaxone 2 g/day and vasoactive drugs were initiated. Chest radiography and urine exams were normal, and abdomen-CT did not show signs of abscess or ascitis. His blood culture grew *Streptococcus anginosus,* only resistant to clindamycin and erythromycin. The antibiotic was continued for 7 days and the patient quickly improved and was discharged. In August 2014, he returned presenting only fever. His blood culture revealed group C beta-hemolytic *Streptococcus*, only resistant to clindamycin and erythromycin, and *Escherichia coli* susceptible to all antibiotics tested. Full abdomen-CT, chest-CT, transesophageal ecochardiography, colonoscopy and a positron emission tomography did not show any signs of infection. Upper digestive endoscopy did not show any complications at the site of the cyanoacrylate injection. The patient was treated for 4 weeks of IV ceftriaxone and discharged. In September 2014, he returned, presenting with severe sepsis and blood cultures positive for *Enterococcus faecalis* susceptible to ampicillin and *E. coli* susceptible to multiple antibiotics. An abdominal CT was performed and a radio-opaque material was detected within the perigastric and cardiophrenic varicose veins corresponding to cyanoacrylate embolization. Ampicillin (12 g per day) and ceftriaxone were maintained for 4 weeks. Due to the recurrent episodes of bacteremia, the patient was discharged using of amoxicillin–clavulanate (500/125 mg) three times-a-day on a continuous basis. Since then, the patient remains free of episodes of bacteremia.

## Literature review

Besides the present case, six other cases of recurrent bacteremia following variceal sclerosis with *N*-butyl-2-cyanoacrylate were reported in the English language literature. The characteristics of the seven cases are shown in Table 1 [2, 4, 6, 14]. All patients had portal hypertension and the indication of injection of cyanoacrylate was bleeding from gastric varices.

**Table 1:** Characteristics of seven patients with recurrent bacteremia following the injection of *N*-butyl-2-cyanoacrylate for the treatment of bleeding gastric varices

	Case number	Gender	Age (years)	Underlying diseases	Time from application of *N*-butyl-2-cyanoacrylate to beginning of symptoms	Number of episodes with positive blood culture	Microorganism isolated from blood culture	Findings of the investigation	Treatment	Outcome	Long term antimicrobial secondary “prophylaxis”
Wahl P, 2004 [14]	1	Male	60	Alcoholic liver cirrhosis + portal hypertension	3.5 months	3	1st episode: *Propionibacterium acnes* + *Actinomyces odontolyticus* 2nd episode: *Propionibacterium acnes Streptococcus anginosus* + *Prevotella species* 3rd episode: *Prevotella oralis*	No significant findings	1st episode: IV^b^ ceftriaxone (duration not reported) 2nd episode: amoxicillin/clavulanate (duration not reported) 3rd episode: moxifloxacin (duration not reported) + surgical debridement of cyanoacrylate plug	Death	No
	2	Male	57	Alcoholic liver cirrhosis + portal hypertension	12 months	2	*Enterobacter aerogenes*	Inflammation around cyanoacrylate plug	IV^b^ ceftriaxone (duration not reported)	Cure	Yes (oral ciprofloxacin for 3 months)
Wright G, 2009 [6]	3	Male	38	Alcoholic liver cirrhosis + portal hypertension	6 months	3	ESBL^a^-producing *E. coli*	Cyanoacrylate embolism to inferior vena cava and left renal vein	IV^b^ ertapenem for 6 months	Cure	No
Galperine T, 2009 [4]	4	Male	69	Idiopathic portal thrombosis + portal hypertension	6 months	2	*Micromonas micros*	Abscess contiguous to cyanoacrylate material	IV^b^ clindamycin + impenem for 2 weeks	Cure	Yes (oral Cefuroxime for 3 months)
	5	Male	46	Hepatitis B cirrhosis + portal hypertension + HIV	2 weeks	3	*Klebsiella pneumoniae*	Cyanoacrylate embolism to spleen and left renal vein	IV^b^ piperacilin/tazobactam for 2 weeks	Cure	Yes (oral amoxicillin/clavulanate for 6 weeks and then IV^b^ ceftriaxone for 3 months)
Reuken PA, 2012 [2]	6	Male	77	Alcoholic liver cirrhosis + portal hypertension	3 months	2	ESBL^a^-producing *E. coli*	Incomplete obliteration of gastric varices	IV^b^ imipenem (duration not reported) + surgery with cardia resection + spleno-renal shunt	Death	No
Present case	7	Male	43	Auto immune hepatitis and cirrhosis + portal hypertension + diabetes mellitus	2 months	3	1st episode: *Streptococcus anginosus* 2nd episode: Group C beta-hemolytic *Streptococcus* + *E. coli* 3rd episode*: E. coli* + *E. faecalis*	Cyanoacrilate embolism to perigastric and cardioprhenic veins	1st episode: IV^b^ ceftriaxone for 7 days 2nd episode: IV^b^ ceftriaxone for 4 weeks 3rd episode: IV^b^ ampicillin + ceftriaxone for 4 weeks	Cure	Yes (amoxicillin/clavulanate- indefinitely)

^a^ *ESBL* extended spectrum beta-lactamase

^b^ *IV* intravenous

The average age of the patients was 55.7 years and the median time from the endoscopic procedure to the first episode of bacteremia was 105 days (range 14–365). The average number of episodes of bacteremia per patient was 2.5.

All cases, after extensive investigation, showed no other focus of infection. In one patient, the same microorganism from the blood culture grew from the cyanoacrylate thrombus after a specimen was collected using endoscopy [2].

## Discussion

*N*-butyl-2-cyanoacrylate is a liquid glue that can be used in several types of human and veterinary procedures, such as patch fixation for hernia repair [7], uterine artery embolization for leiomyomas and placenta increta [8], treatment of renal arteriovenous malformation [9] and for obliteration of gastric varices [1–6, 10–14]. Recurrent bacteremia has been described only for this last procedure.

Around 90 % of patients develop transient fever after *N*-butyl-2-cyanoacrylate injection [14], however the incidence of bacteremia after the procedure is not clear. Chen et al. observed that the frequency of bacteremia in the cyanoacrylate group was higher than in the group under upper endoscopy without cyanoacrylate injection (15/47 vs. 1/47, *p* < 0.0001) [11]. Furthermore, high volume of blood transfusion and Child-Pugh score were factors associated with the occurrence of bacteremia [11]. Jun et al. observed a frequency of 0.4 % of bacteremia and a Brazilian study did not showed differences in incidence of bacteremia between band ligation and cyanoacrylate injection [3]. A prospective study with 55 patients only reported bacteremia after injection for treatment of bleeding gastric varices. Elective cyanoacrylate injection for nonbleeding gastric varices was not associated with any episodes of bacteremia [13].

Recurrent bacteremia, however, is a very rare event with only seven cases described. All patients reported underwent cyanoacrylate treatment for bleeding gastric varices. Because of the rarity of the condition, recurrent bacteremia associated with injection of cyanoacrylate should be a diagnosis of exclusion and other more common diagnoses, such as spontaneous bacterial peritonitis, must be investigated. New diagnostic tools, such as F-18 FDG PET/CT, may be useful in the diagnosis of infected cyanoacrylate plug [12], but require more studies.

In six of the reported cases including this one, complications associated with cyanoacrylate, such as abscess [4] or embolism [4, 6], were found. Two cases of persistent (but not recurrent) bacteremia reported showed incomplete obliteration of gastric varices, possibly facilitating bacterial translocation. In these cases, fever subsided after total obliteration [5].

Most recurrent episodes of bacteremia were caused by the same microorganism [2, 4, 6], except for the present report and one other [14], in which multiple bacteria were implicated. All the microorganisms isolated from blood cultures in all reports were commensals from the oral or gastrointestinal flora. Thus, migration of bacteria from the gastrointestinal lumen to blood vessels was probably facilitated by the cyanoacrylate plug. Polymerization of cyanoacrylate has been described to form a large conglomerate that may protrude intraluminally and provide an ideal route for bacterial invasion [10, 14].

We believe that after an episode of bacteremia following the injection of *N*-butyl-2-cyanoacrylate for the treatment of bleeding gastric varices, in which there is no evidence of the source of infection, long term secondary prophylaxis with antimicrobials should be considered due to the gravity of these bloodstream infections.

In conclusion, recurrent bacteremia associated with previous endoscopic treatment with *N*-2-butyl-cyanoacrylate is very rare, but should be suspected in patients in which extensive investigation fails to show another focus of infection. Complications associated with cyanoacrylate must be ruled out. Secondary prophylaxis should be considered in these patients after the first episode of bacteremia.

## Consent

Written informed consent was obtained from the patient for publication of this case report and any accompanying images.
